# Understanding the role of electrostatic force, van der Waals force, and osmotic pressure in retinal function and barrier integrity

**DOI:** 10.1186/s40942-025-00643-y

**Published:** 2025-02-19

**Authors:** Khayry Al-Shami, Jafar Shatnawi, Khaled Qasagsah, Salman Almurabi, Ghayda’ Shatnawi, Tasnim Darawsheh, Shahed Karaja

**Affiliations:** 1 Department of Clinical Medical Sciences, Faculty of Medicine, Yarmouk University, Irbid, Jordan; 2 University of Hama Faculty of Human medicine, Hama, Syria

**Keywords:** Retinal epithelium, Outer segment photoreceptor, Electrostatic Force, Van Der Waals Force, Osmotic pressure

## Abstract

The retina’s intricate interplay of forces and structures, with a focus on the retinal pigment epithelium (RPE) and photoreceptors, is essential for retinal health and function. Among these forces, electrostatic forces play a crucial role, working alongside van der Waals forces and oncotic pressure to maintain the retina’s attachment to the RPE and ensure the integrity of the blood-retina barrier (BRB). The composition of the interphotoreceptor matrix (IPM), influenced by molecules like Retbindin secreted by rod photoreceptors, further modulates these forces, affecting processes like visual pigment regeneration and metabolite exchange. In the context of retinal tissue engineering and new technologies for support and cells-based treatments, electrostatic forces are harnessed to optimize nutrient supply to transplanted RPE cells by reducing pore size in electrospun polymer membranes. Scaffold-based strategies for retinal repair also utilize electrostatic, hydrophobic, van der Waals, and hydrogen bonding forces to enhance cell adhesion and growth, mimicking the basement membrane. Understanding the complex dynamics of these forces in retinal-RPE interactions holds promise for innovative treatments for retinal disorders, emphasizing the intricate balance between electrostatic forces, van der Waals forces, oncotic pressure, and more. These insights open exciting avenues for research and therapeutic interventions in ophthalmology. Additionally, van der Waals forces are explored in the context of cell adhesion, and their potential role in retinal health is discussed, particularly in relation to melanin’s protective properties against blue light-induced damage. Tissue engineering approaches, both scaffold-free and scaffold-based, are discussed, highlighting the importance of physical surface treatments and adhesive forces in preserving engineered RPE tissue. Overall, this abstract provides a comprehensive overview of the multifaceted role of electrostatic and other forces in retinal biology and their implications for future research and clinical applications in ophthalmology.

## Introduction

The intricate interplay between various forces and structures within the retina, with a focus on the retinal pigment epithelium (RPE) and photoreceptors, is pivotal for maintaining retinal health and functionality. Electrostatic force, stemming from the interaction of electric charges among molecules and surfaces, is one of the central forces in this complex orchestration. It works in tandem with other forces like van der Waals forces and oncotic pressure to sustain the delicate equilibrium that secures the retina’s attachment to the RPE and ensures the proper operation of the blood-retina barrier (BRB) [1]. Within this intricate context, the retina, a multifaceted tissue responsible for light-to-signal conversion, houses a diverse array of cell types, including photoreceptors, bipolar cells, ganglion cells, and the indispensable RPE. Positioned between photoreceptors and the choriocapillaris, the RPE performs critical vision-related functions such as outer segment recycling and nutrient transportation. At the core of the interaction between the retina and RPE lies the subretinal space, an extracellular region enveloping the photoreceptors, where electrostatic forces, especially notable on the RPE’s apical microvilli, act as both anchors and protectors, thwarting the detachment of these two layers [2]. Additionally, the interphotoreceptor matrix’s (IPM) composition and properties, influenced by molecules like Retbindin secreted by rod photoreceptors, further modulate these forces, impacting various vital processes like visual pigment regeneration and metabolite exchange within the IPM. In the realm of retinal tissue engineering and regenerative medicine, electrostatic forces come into play, particularly in experiments involving frame-supported ultrathin electrospun polymer membranes [3]. These experiments aim to optimize electrostatic forces to enhance nutrient supply to transplanted RPE cells in the subretinal space by reducing pore size, thereby facilitating cell retention or the formation of multicellular aggregates. Furthermore, scaffold-based strategies for retinal repair employ physical surface treatments harnessing electrostatic, hydrophobic, van der Waals, and hydrogen bonding forces to boost cell adhesion and growth. These methods emulate the basement membrane and provide a three-dimensional environment conducive to RPE tissue regeneration [4]. As our understanding deepens regarding the intricate dynamics of retinal-RPE interactions and the role of electrostatic forces, new insights emerge into the forces governing retinal health. These revelations hold promise for innovative approaches in the treatment of retinal disorders, emphasizing the intricate balance between electrostatic forces, van der Waals forces, oncotic pressure, and more. This intricate interplay paints a captivating picture of the complex mechanisms underpinning ocular biology, opening exciting avenues for research and therapeutic interventions in the field of ophthalmology.

### Electrostatic force

**Fig. 1:**
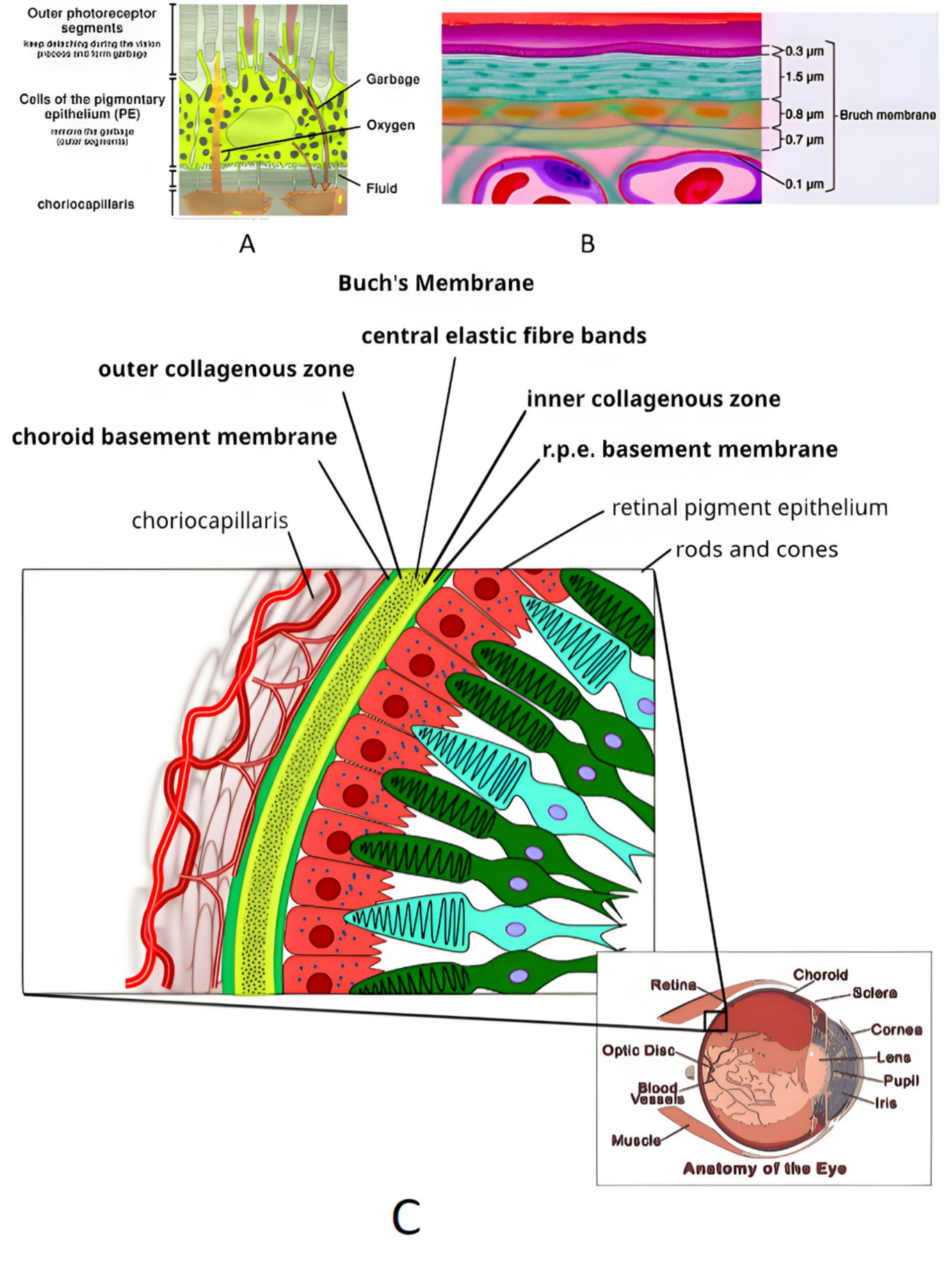
The retinal pigment epithelium (RPE) and the Bruch membrane have a significant relationship. (**A**, **C**) The Bruch membrane is a barrier separating the RPE from the choriocapillaris. It is important to observe the intricate interdigitation of the apical processes of the RPE with the photoreceptor outer segments, as well as the infoldings of the basal surface. (**B**) The diagram illustrates the thickness of the various layers of the Bruch membrane, starting from the top: the basement membrane of the RPE, the inner collagenous layer, an elastic layer, an outer collagenous layer, and the basement membrane of the choriocapillaris. The apical processes of the RPE are identified as APRPE

Tight junctions encircle RPE cells near their apical side, creating a division in their membrane between the basal (basolateral) and apical regions. The basal membrane and tight junctions are essential for establishing polarity, which prevents the passage of water and ions and forms the outer blood-neural retinal barrier [5, 6](see illustration A, C,B Fig. 1). The apical membrane of the retinal pigment epithelium (RPE) extends numerous villous and sheet-like processes that tightly envelop and encase sections of the outer segments of both rods and cones. Despite these two membranes’ distinct environments and contrasting anatomical and functional characteristics, they work together to transport metabolites, water, and salts between the blood and the subretinal space. The subretinal space is the extracellular region surrounding the photoreceptors [7]. The apical surface of RPE cells is adorned with elongated microvilli that establish an intricate anatomical connection with the outer segment of the photoreceptor [8]. The elongated microvilli on the apical surface of RPE cells extend and interlock with the tips of the photoreceptor cells’ outer segment, composed of stacked cells. These microvilli serve important functions, such as facilitating the phagocytosis and renewal of rods and providing frictional resistance against separation forces like withdrawal or electrostatic force, preventing the detachment of the two layers. These forces are also influenced by the composition and properties of the interphotoreceptor matrix that lies between them, as well as the flexibility and adaptability of the RPE microvilli. The outer retina is particularly susceptible to detachment at points where structural weaknesses exist concerning adjacent layers, as the separation forces overpower the physiological adhesive forces of the retina, including van der Waals forces, oncotic pressure, and electrostatic forces [9–11].

In a frame-supported ultrathin electrospun polymer membrane experiment involving the transplantation of retinal pigment epithelial cells, elucidate how using electrostatic force optimizes the provision of nutrients to the transplanted cells within the subretinal space. This approach aims to enhance the therapeutic impact of the transplantation by reducing pore size, thereby promoting the retention of cells or the formation of multicellular aggregates within the cell suspension [12, 13]. Another study talking about methods to regenerate the impaired retina by delivering photoreceptor precursor cells and pigment epithelium to the subretinal space. Scaffold delivery strategies enhance cell survival and differentiation. These methods include: (1) Solvent casting (2) Particulate leaching (3) Phase separation (4) Freeze drying (5) Microfabrication (6) Electrospinning(Look at Table 1) [14–22].

**Table 1:** Techniques for fabricating polymer films and scaffolds

Technique	Description
Solvent Casting [1]	A common method used for the fabrication of polymer films and scaffolds. Involves dissolving a hydrophobic polymer in a solvent, casting it into a mold, and evaporating the solvent, leaving behind a thin, nonporous film. Used for subretinal space scaffolds.
Particulate Leaching [2]	Combines a polymer solution with a solvent-insoluble porogen to develop porous scaffolds. After solvent evaporation, the porogen is dissolved in an appropriate solvent (e.g., water), resulting in a porous sponge or foam with an open network.
Phase Separation [3]	A technique used to fabricate porous fibrous scaffolds, producing structures with porous networks.
Microfabrication [5]	Controls the physical and chemical architecture of materials on a microscale and nanoscale. Techniques include soft lithography and photolithography, where UV light exposure through a mask creates specific architectures or patterns.
Photolithography [5]	A microfabrication method where UV light exposure through a mask dictates the exposure pattern to fabricate precise architectures.
Electrospinning [6]	Utilizes high electrostatic forces to produce polymer fibers ranging from nanometers to hundreds of microns, suitable for various scaffolds.

Another research study indicates the presence of a specific Extracellular Riboflavin-binding Protein, known as Retbindin, which is secreted by rod photoreceptors into the inter-photoreceptor matrix (IPM). The adhesion between the retinal pigment epithelium (RPE) and the photoreceptor layer is facilitated by electrostatic forces that help retain this protein within the IPM. Retbindin acts as a carrier between the two layers and plays a crucial role in various processes taking place within the IPM, including visual pigment regeneration, phagocytosis of outer segments by the RPE, exchange of metabolites, and attachment of the neural retina to the RPE. Precise regulation of these processes is vital for maintaining photoreceptors and properly functioning phototransduction [23, 24].

### Tissue engineering and common eye diseases

The first to third most common eye diseases that may be addressed through TE include corneal diseases (prevalence rate approximately 1 in 500), age-related macular degeneration (AMD) (affecting about 10% of individuals over 65), and diabetic retinopathy (prevalence rate around 30% among diabetics), all of which present opportunities for innovative therapeutic interventions [25]. TE presents significant potential for the treatment of various ophthalmopathies through the regeneration or repair of damaged ocular tissues. The regeneration of corneal stroma using biomaterials and stem cells stands as a main goal of TE because it restores both function and transparency of damaged corneas. When treating keratoconus, health providers use collagen cross-linking and grafting methods to build up corneal strength. Scientists have demonstrated successful corneal epithelium restoration with improved visual results through growing limbal stem cells on suitable scaffold structures in patients with limbal stem cell deficiency. Engineered grafts assist in the wound healing of persistent epithelial defects by helping the corneal surface mend. Scientists have initiated TE strategy research to develop solutions for retinal disorders, especially age-related macular degeneration (AMD) [26]. Medical scientists work on developing retinal pigment epithelium (RPE) patches from stem cells to replace injured retinal tissue. Engineered retinal patches serve as an effective solution for both retinal reattachment needs and support of retinas when detachment occurs. The development of these technologies is vital for bringing back sight in patients who suffer from degenerative retinal diseases.Versions of TE are developing glaucoma treatments through implant creation, which regulates intraocular pressure and aids in optic nerve fiber regeneration [27]. Local drug-delivery systems based on tissue engineering give medical professionals a tool to directly target uveitis inflammation in the uvea. Finally, TE approaches are also focused on regenerating the optic nerve using nerve grafts or scaffolds to support neuronal growth. The field of eye TE has emerged as a significant area of clinical practice and research, offering innovative strategies that collectively promise to enhance the quality of life for patients with various ocular conditions [28].

### Tissue engineering in regeneration

The life-sustaining capability of human beings emerges from their natural ability to restore themselves after physical damage. Changes in TE and regenerative medicine technologies have significantly progressed during the last three decades. The primary objective of this field becomes tissue regeneration without the complete removal of damaged tissues. Tissue engineering developers establish biological materials to restore original tissue functions and boost their operational ability [29]. The National Science Foundation (NSF) used Granlibakken, California, as the venue for their workshop, which brought forward the term ‘tissue engineering.’ The three categories of TE practice include (1) isolated cell implantation that consists of cell transplantation, (2) growth agent administration to enhance cell multiplication, and (3) embedding cells within scaffolds to prompt ECM production or secretion. The process of cell placement onto extracellular scaffolds represents the standard TE technique that leads to implantable substrates. Cell adhesion, together with cell proliferation and cell differentiation, experiences direct influence through the primary interaction site located on scaffold surfaces [30]. The important opportunity exists to vascularize large scaffolds to allow sufficient delivery of minerals, nutrients, oxygen, and growth factors that support tissue regeneration. The scaffold employs special traits for attracting target cells before they begin cell division as well as specialization toward forming new tissues inside the scaffold framework. The scaffolding structure will break down eventually while the newly formed tissue remains the sole element present after the deconstruction process. Tissue-engineered nerve grafts (TENGs) have been identified as a viable alternative for autologous nerve grafts, which are considered as the most effective method for repairing peripheral nerves. Henceforth, cell-based therapies are regarded as a preferred approach in tissue regeneration. Restoring the functionality of impaired bodily tissues through the repair or reconstruction of faulty structures has long been a goal of medical science. TE has emerged as a field dedicated to tackling this very challenge [31].

### Safety concerns for scaffold biomaterials

Scaffold biomaterials used in TE and regenerative medicine need thorough assessment on safety grounds through multiple essential criteria. The basic need for biocompatibility assurance in scaffold materials exists to stop harmful immune effects and toxic reactions. The scaffold degradation products need to be nontoxic substances that produce no inflammatory reactions in the body. The scaffolding mechanisms require proper mechanical attributes because they should have enough structural strength to maintain tissue development until it reaches maturity. Protecting sterility remains crucial because it stops post-implantation infections from happening yet scaffolds require lasting stability systems to reduce likely chronic complications so regulators need comprehensive adherence requirements before clinical use begins. The scaffold design must allow proper cell-cell contacts, which promote adhesion and differentiation but prevent unwanted cell errors [32]. Biomaterial safety evaluations must be complete because their origin creates disease transmission concerns and ethical implications and requires detailed assessments of environmental impact for ecological protection while leading to individual testing of scaffold safety because patients exhibit different responses. The implementation of scaffold biomaterials in clinical practice requires proper investigation of safety aspects because ocular diseases specifically depend on scaffold biocompatibility combined with sterility standards and mechanical requirements for achieving beneficial treatment results and reducing tissue repair complications [33].

### Perspective for ocular tissue engineering

Regenerative medicine features ocular TE as its essential area because it aims to rebuild damaged ocular structures and restore sight by using innovative biomimetic scaffold and cellular therapy techniques. Research shows that scientists have made progress in creating advanced biomaterials that demonstrate both optimal biocompatibility features together with biodegradability capability as well as mechanical properties that fit the sensitive nature of the eye. The development of procedural methods in ocular medicine now combines stem cell approaches that use induced pluripotent stem cells (iPSCs) with growth factors to improve tissue repair functions. 3D bioprinting, together with nanotechnology strategies, are fast-emerging fields that enable the creation of intricate tissue structures that resemble natural human tissues, which improves TE methodology accuracy [34]. Research documents show that safety considerations must be addressed first because immune rejection risks and infection dangers represent barrier-to-entry problems for the clinical implementation of these technologies. Strategic management of regulatory standards together with ethical examination of biological materials usage requires specific attention during operational processes. Advanced techniques for ocular TE demonstrate strong potential benefits for creating better treatment approaches to various eye conditions such as corneal injuries, retinal degeneration, and glaucoma, which will improve clinical results and patient life quality. New research reveals that personalized techniques employing customized scaffolds and delivery systems will help transform medical practices within this field in the future [35].

### The creation of eye tissues requires different cell populations

Various types of cells have been used for conducting eye tissue engineering research. The two primary cell types utilized for conjunctival scaffolds were conjunctival mesenchymal stem cells (CJMSCs) and rabbit conjunctival epithelial cells (rCjECs), and researchers managed to successfully cultivate both types. Human adipose mesenchymal stem cells (hADSCs), limbal stromal stem cells (LSSCs), keratocytes, rabbit corneal limbal epithelial cells, limbal epithelial progenitor cells (LEPC), limbal mesenchymal stromal cells (LMSC), and limbal melanocytes (LM), Rabbit Corneal stromal cell, human embryonic stem cells-corneal epithelial cells (hESC-CEC), rabbit corneal epithelial cells, corneal epithelial cells (CEC), human corneal fibroblasts (HCFs) cells, human corneal endothelial cells (hCEC), human limbal epithelial cells (hLEC), Human keratocytes (HKs), human corneal epithelial cells (HCECs), and corneal keratocyte cell were the main cellular components employed in the construction of cornea scaffolds and every one of them was grown successfully [36].Adult rabbit lacrimal gland (LG) progenitor cells, Lacrimal acinar epithelial cells of Sprague-Dawley rats, porcine LG epithelial cells, purified rabbit lacrimal gland acinar cells (pLGACs), and LG epithelial cells were found as cells seeded in lacrimal gland scaffolds. Mouse retinal progenitor cells (mRPCs), retinal ganglion cells (RGCs), retinal pigment epithelium cells (RPE), human fetal RPE (hfRPE), human embryonic stem cells, human-induced pluripotent stem cells (hiPSCs), and CJMSCs are the main cells used effectively in retinal scaffolds. Scientists use neurotrophin-3-overexpressing Schwann cells as well as dorsal root ganglion (DRG) neurites and RGCs together with retinal ganglion cell progenitors (RGCP) and chick dorsal root ganglia and a mouse neuroblastoma cell line for effective optic nerve scaffold development [37].

### Tissue engineering scaffolds are being utilized in present clinical trials

Two eye scaffold-related clinical trials exist in the current field. The clinical study implemented crosslinked RHC implants based on carbodiimide processing to solve the worldwide problem of donated cornea shortages. The new corneas existed for four years while demonstrating proper development into their host environments without any signs of rejection. The patients receiving these neo-cornea treatments bypassed the mandatory immunosuppression therapy normally needed for individuals who receive donor corneal transplants. Research did not observe the migration of inflammatory dendritic cells within the implant area. The migration of dendritic cells into central cornea regions occurred in donor cornea recipients, although they received immunosuppressive medications during the time of these detections. These observations subsequently led to rejection outcomes. The four-year observation period exhibited ongoing cell population of nerves and stromaria leading to micro-architectural characteristics that approached those of normal healthy corneas. During a four-year study period, patients who received implantation received a 20/54 average visual acuity improvement [38]. The visual ability of these patients substantially improved through their ability to see an additional 5 Snellen lines when tested on an eye chart. Visual acuity enhancements occur when medical professionals develop materials with better shape retention qualities. The second clinical trial in this area was for a corneal scaffold. This clinical trial uses a nano-structured fibrin agarose corneal substitute that incorporates allogeneic cells, successfully replicating the mechanical, optical, and biological properties of the anterior human native cornea. The still-ongoing clinical trial performed in ten hospitals in Spain is a controlled, randomized, open-label study covering both phase I and phase II. This is a single-arm, uncontrolled interventional study designed to evaluate the safety and efficacy of a bioengineered human corneal substitute in adults with severe trophic corneal ulcers resistant to standard medical treatment or who have developed complications due to previous ulcers. Also, the clinical trial should provide clinical evidence for the efficacy of the intervention. Determination of safety and practicability will be based on adverse event assessment, device condition, signs and symptoms related to infection, and determination of induced neoangiogenesis. Such factors represent the main outcomes of the current study. Measurement of study endpoints will be followed for a total of 24 months and shall include a sum of 27 post-implant assessment visits. These are visits that are done at increasingly reduced intervals within the follow-up period. In the end, the application of scaffolds in TE methods is showing tremendous potential for ocular tissue regeneration. By harnessing the power of biomaterials science and stem cell technology, active researchers pioneer new treatments with the potential to change the face of ophthalmology and improve outcomes in patients with many eye conditions [39, 40].

### Oncotic pressure

Normal human plasma has a concentration of protein of approximately 7 g/dl [41], Albumin, which has a molecular weight of 69,000 Kd is the main protein responsible for maintaining colloid oncotic pressure. It makes up around 69–75% of this pressure. In contrast, Fibrinogen and globulins have molecular weights that vary between 45,000 Kd and 1,000,000 Kd and account for the rest of the active proteins [42].

Oncotic pressure, also known as colloid osmotic pressure, is generated by proteins, primarily albumin, and it induces the movement of fluids back into the capillaries. In contrast, hydrostatic pressure acts as the opposing force, pushing fluids out of the capillary. The retina, especially the macula, is highly susceptible to oxidative stress due to its elevated metabolic demands, which can result in damage to central vision [43].

To safeguard against such damage, the microenvironment of the retina needs to be separated from the systemic circulation by the Blood Retina Barrier (BRB). The primary function of the BRB is to preserve the integrity of the retina [2].

The blood-retina barrier (BRB) is like a protective wall in the eye. It’s made up of tiny blood vessels in the inner part of the eye and cells called retinal pigment epithelium (RPE) that keep the blood in the body from reaching the retina. The inner part of this barrier is called the inner blood-retina barrier (iBRB), and it’s made of special cells on the tiny blood vessels in the inner part of the eye. The outer part is called the outer blood-retina barrier (oBRB), and it’s made of cells in the RPE that separate the retina from the blood vessels outside the eye [44].

The human retina has six main types of cells in different layers. These include photoreceptors (rods and cones) in the outer layer, bipolar and amacrine cells in the inner layer, retinal ganglion cells in the innermost layer, and other cells called horizontal and Müller cells. The ganglion cell axons go to the back of the eye and come together to make the optic nerve [45].

**Fig. 2:**
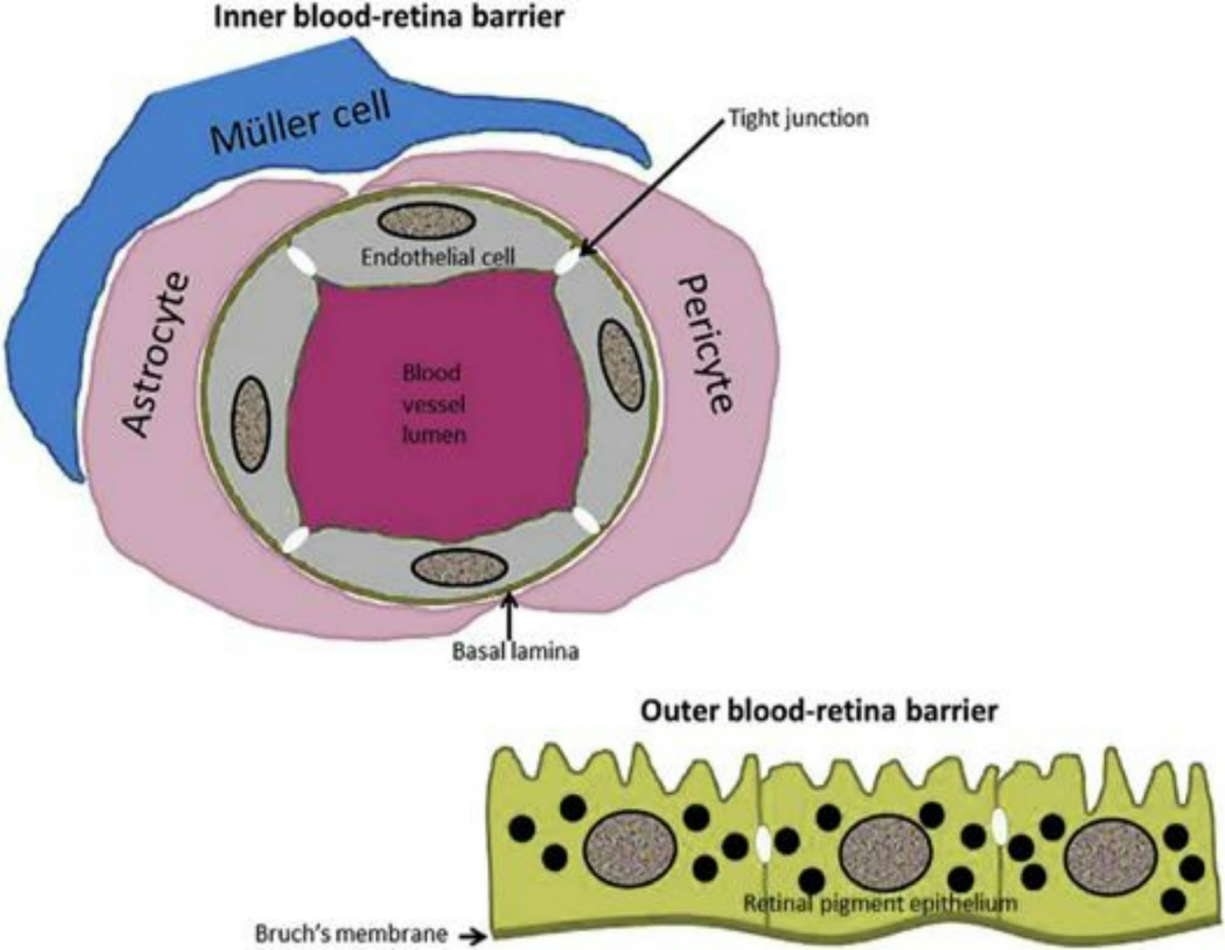
Diagram Illustrating the cells and structures that make up the inner and outer blood-retina barriers

The blood-retinal barrier (BRB) is like a protective shield for the retina, much like the blood-brain barrier (BBB) is for the brain. It keeps harmful chemicals and big molecules in the blood from reaching the retina by controlling what can pass through. The BRB has two parts: an inner layer and an outer layer (Fig. 2– on RPE figure, please remove from inside the cell the name “RPE”, replace to outside or remove, please. Furthermore, be clear where are the “blood” explained external blood barrier.) [2].

**Fig. 3:**
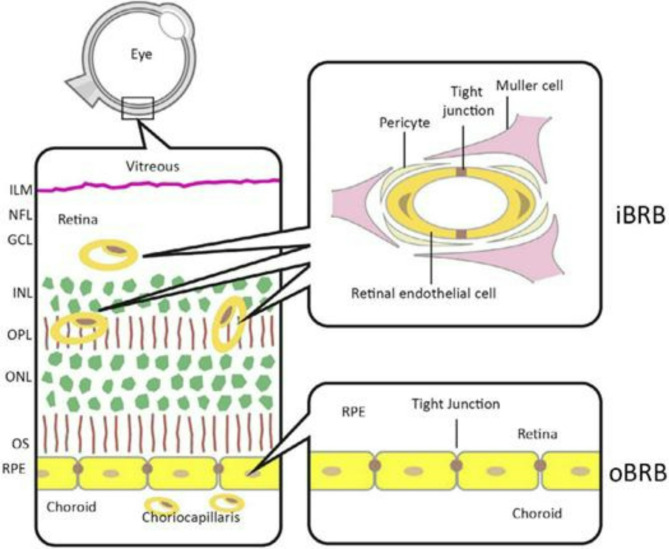
The positioning of the Inner and outer blood-retina barriers. The I nner blood-retina barrier (iBRB) Is created by the endothelial cells that line the blood vessels in the inner part of the retina. These blood vessels supply the retina up to the outer plexiform layer (OPL), and the layer of photoreceptor cells remains without blood vessels. The outer blood-retina barrier (oBRB) is formed by the retinal pigment epithelial cells (RPE) and controls the movement of substances between the retina and the choroid. In this context, we also have the Inner limiting membrane (ILM), ganglion cell layer (GCL), inner nuclear layer (INL), outer nuclear layer (ONL), and outer segments (OS)

The choriocapillaris has tiny openings and plays an important role in providing nutrients and getting rid of waste from the outer parts of the retina, which includes the retinal pigment epithelium (RPE) and photoreceptors (PRs) Fig. 3 [46].Bruch’s membrane (BM) is found in the space between the basement membrane of the choriocapillaris and the basement membrane of the RPE. It’s made up of two collagenous layers on the outside, with an elastic layer in the middle [47].The BM permits the selective passage of molecules based on their size, allowing smaller molecules to pass while preventing larger ones from diffusing through [48].Other functions involves stabilization of the RPE layer and absorption of physical stress forces [49]. The RPE is a layer of cells shaped like hexagons, containing pigments, and it sits right under the neural retina. In the RPE, the tight junctions (TJs) are found at the top surface, and they are mainly responsible for keeping the outer blood-retina barrier (oBRB) strong and intact [50].Numerous Microvilli stretch out from the top surface of the RPE and wrap around the outer segments of photoreceptors (POS). This wrapping increases the contact surface area with POS by about 30 times, which encourages more contact between the cells [51].

When the retinal pigment epithelium (RPE) is damaged, whether it’s due to physical injury, photocoagulation, or exposure to sodium iodate, it can lead to a faster removal of saline fluid from the area beneath the retina [52, 53].regulating how molecules enter and leave the retina is essential for maintaining a healthy balance in the retina. The structures of the blood-retinal barrier (BRB) play a vital role in this regulation. The transport of molecules across the RPE can occur through either transcellular or paracellular routes. Osmotic pressure plays a role in paracellular transport, At the outer BRB, most of the transport happens transcellularly, as there’s greater resistance when molecules try to pass paracellularly [54, 55].

**Fig. 4:**
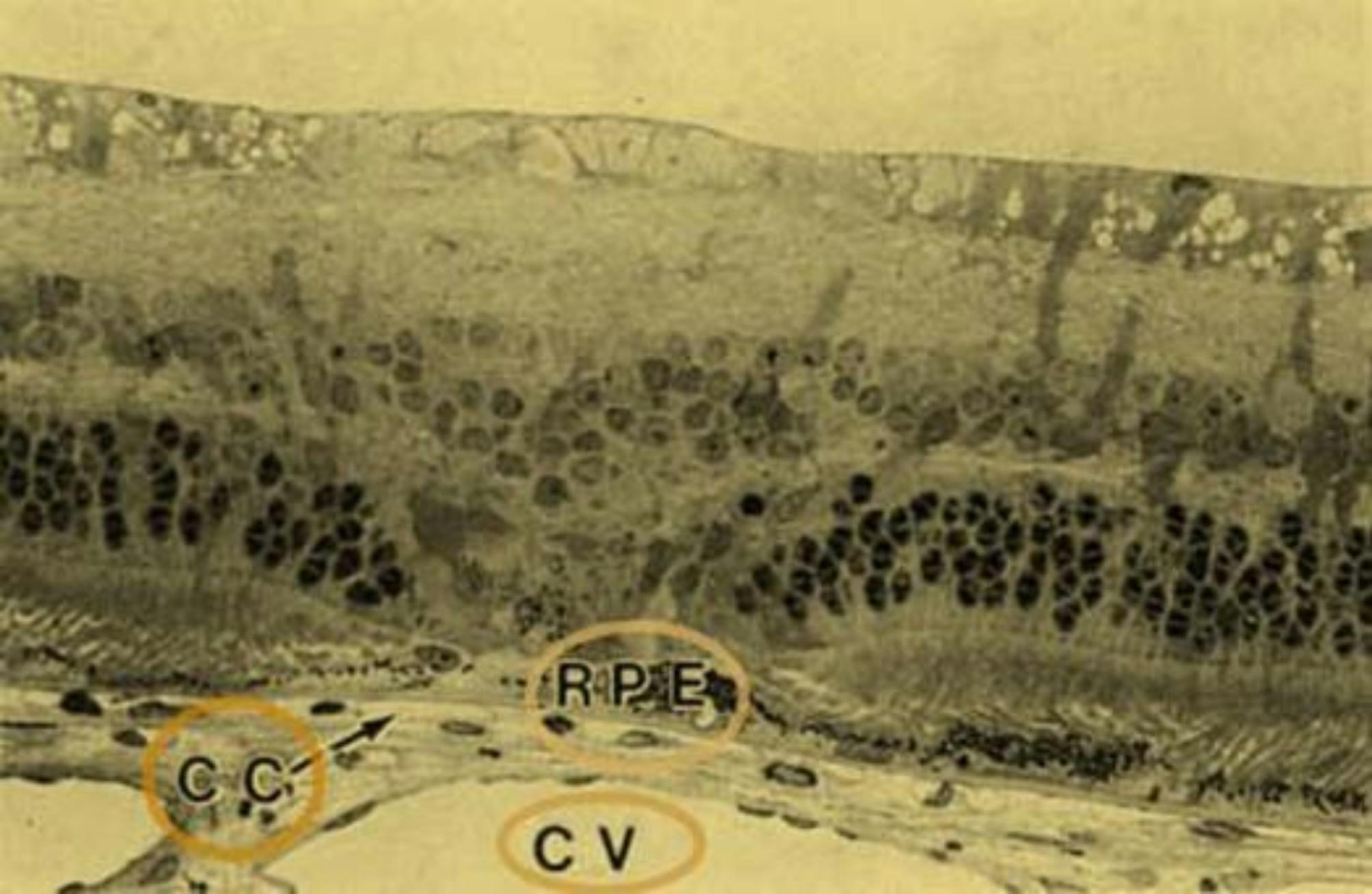
Light micrograph of a 2-week old argon laser burn in a pigmented rabbit retina

It’s not just the structures inside cells and the proteins in tight junctions (TJ) that affect the blood-retinal barrier (BRB) and how the environment in the retina is regulated. The forces that move fluid and ions across the retina also play a role in understanding what happens in various retinal diseases when the BRB weakens and causes swelling [56].In a healthy eye, the pressure inside the eye pushes water into the spaces between the retinal layers. However, the choroidal circulation creates an opposing pull due to differences in concentration, which draws water back into the blood vessels. This process keeps the retina dry and maintains its position [57].Adhesion between the retina and RPE involves two forces: mechanical and metabolic [51]. Mechanical forces can be categorized into those acting outside and inside the subretinal space (SRS). External mechanical forces include the pressure exerted by the vitreous and fluid, while internal mechanical forces involve the interdigitations between RPE microvilli and photoreceptors, as well as the matrix material between the SRS and RPE [58–60]. Glucose and essential nutrients primarily reach the retina by passing through cells, while water and electrolytes mainly move from the area beneath the retina through mechanisms involving aquaporin 1 and 4. The energy for this process is supplied by the Na+/K- -ATPase at the top surface of the cells [51, 61].This creates a force which aids anatomical apposition between PRs and the RPE [62]. Tight junctions (TJs) are not a complete blockage, as demonstrated by measurements of TEER. These TJs selectively permit the movement of various ions while blocking others. This selective action creates a concentration difference, which aids in supporting the processes that occur within cells [51].Under usual circumstances, oxygen and nutrients move from the choriocapillaris into the retina, where photoreceptors consume them. Photoreceptors have lots of mitochondria and use a lot of oxygen. In areas where lasers have created scars, the photoreceptors are replaced by glia cells, which have fewer mitochondria and use less oxygen. This makes these laser scars act like openings or windows where oxygen consumption is minimal, allowing oxygen to pass from the choroid through the photoreceptor layer into the inner retina.The following image (Fig. 4.) shows a 2-week-old argon laser injury in a pigmented rabbit’s retina. The outer part of the retina and the retinal pigment epithelium have been treated with photocoagulation, and the photoreceptors are no longer present. However, the inner part of the retina remains relatively undamaged. In the image, “RPE” represents the pigmented retinal pigment epithelium, “CC” is the choriocapillaris, and “CV” represents the large choroidal vessels [63].

**Fig. 5:**
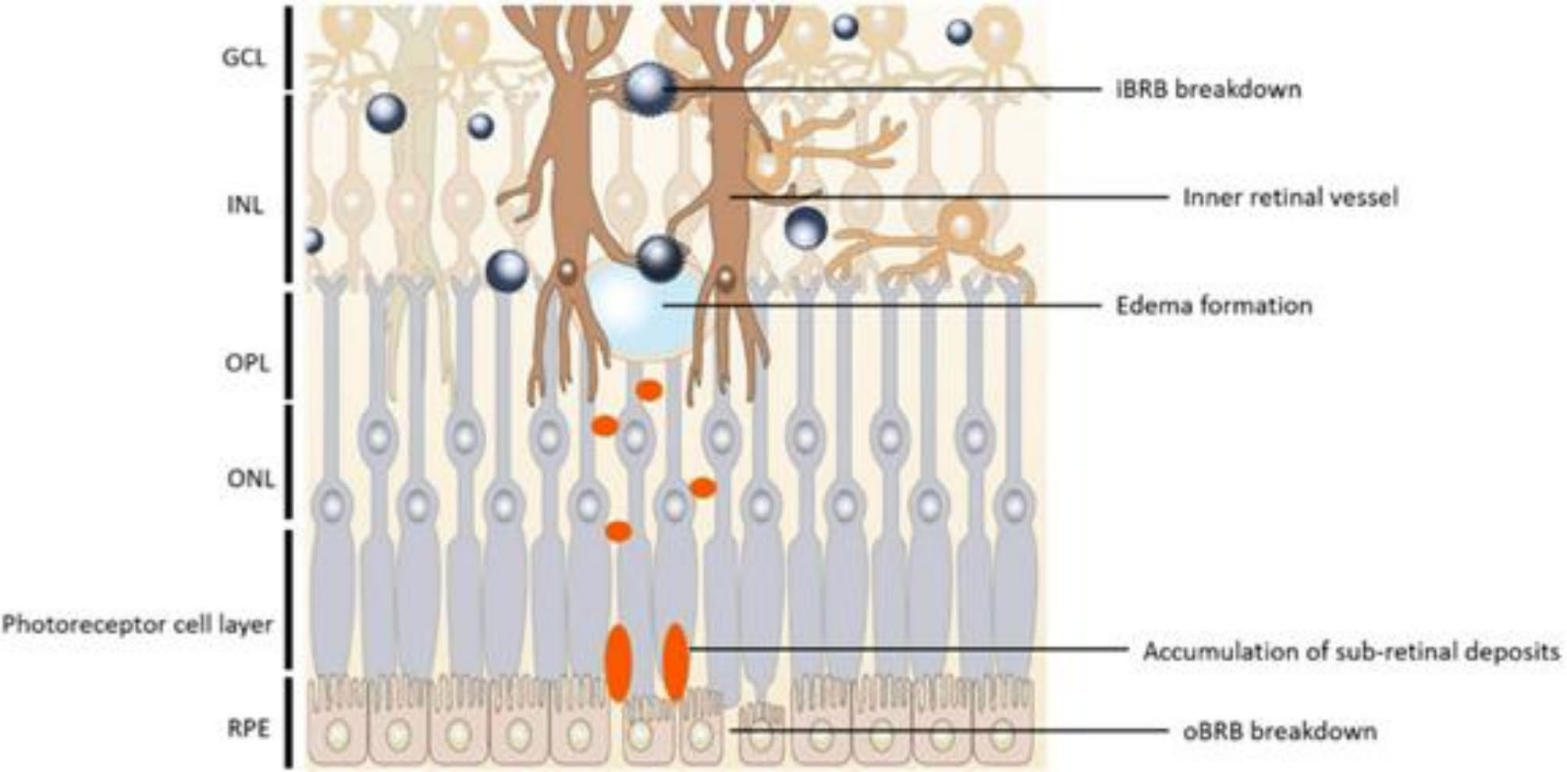
Diagram showing the breakdown of the blood-retina barrier. This breakdown affects the ganglion cell layer (GCL), inner nuclear layer (INL), outer nuclear layer (ONL), and outer plexiform layer (OPL). The outer blood-retina barrier (oBRB) is established by the retinal pigment epithelial cells (RPE)

The factors that stick the retina to the choroid, like intraocular pressure and choroid’s oncotic pressure, along with the fragile connections between photoreceptors (rods and cones) and the RPE. Even the presence of a complex substance known as interphotoreceptor matrix, as well as other metabolic and structural elements, can’t on their own or collectively fully clarify the remarkable characteristics seen in how the photoreceptor layer adheres to RPE cells. The Fact detachment of the retina shortly after death, suggesting that it’s a dynamic process needing energy, is usually linked to intraocular pressure and the oncotic pressure in the choroid’s blood flow. However, even when intraocular pressure is high (as in glaucoma), it doesn’t stop retinal detachment, and the oncotic pressure in the vitreous is nonexistent. The surprising capability of melanin to break apart and then recombine water molecules provides a reasonable explanation for why the retina sticks to the RPE. Firstly, it creates the needed chemical energy to keep the process active, and secondly, this constant splitting and reformation of water molecules generates a suction force [64].The following figures shows different scenarios of breakdown of both of oBRB and iBRB (Fig. 5).

edema can be defined as accumulation of water in the tissues, The development of edema is mostly based on the movement of water between the tissue compartment and the vascular compartment as mentioned before that hydrostatic pressure that makes the water flow into the tissue is opposed by oncotic pressure. However, if oncotic pressure is constant then an increase in hydrostatic pressure increases the chance of having edema while a decrease in hydrostatic pressure decreases the chance of having edema [65].

One of the main complications of Diabetic Retinopathy is the formation of macular edema [66], The blood/retina barrier is formed by is made of the RPE and the endothelium of retinal vessels, macular edema is formed by damage to this barrier. the way through which macular edema is treated is by the administration of inhibitors of carbonic anhydrase [66–71].

Melanin can function as an antioxidant, a weak free radical scavenger and photoscreen [72], in people with dark pigmented eyes have been reported to have lower prevalence of retinal detachment and lower incidence of macular degeneration, it also has been reported that rising in solar radiation also leads to decline in the incidence of uveal melanoma, also has been reported lately that solar radiation can also decrease the risk of having systematic malignant tumors which means that sunlight has two effects on tissue exposed to sunlight and in a indirect way it has protective effect on tissues that weren’t exposed to sunlight [73].

melanin can protect the retina from oxidative damage. anyway, as the person grow; his pigment cells will be repeatedly exposed to high levels of oxygen which may result in decline of the antioxidant properties of melanin, which means that photoreceptors are at high risk of damage as a result for that people with dark pigmented eyes has better chance to resist reactive oxygen species [74].

### Van Der Waals Force

In 1873, van der Waals proposed the existence of forces that attract atoms to explain why gases cannot be compressed easily and how they condense. These attractive forces are present everywhere. The result of imbalances in charges within atoms which then induce a similar effect in neighboring atoms resulting in a weak but observable attraction [75]. Bradley discovered a method to combine these attractions, which was further expanded upon by Hamaker [76, 77]. London developed a quantum theory that aligned with the calculations made by Lennard Jones explaining the characteristics of reactive atoms, like argon [78]. Despite being weaker than covalent bonds, these forces have the advantage of being universal. Can explain the adhesion of simple materials such as paraffins or elastomers that lack electrostatic charges, dipoles or electron sharing capabilities. Various papers and books provide explanations of these forces [79–81]. Considering that van der Waals force plays a role in cell adhesion it is essential to evaluate its impact before considering possibilities, like covalent forces [75]. Certain cells, in the body like blood cells (erythrocytes) can move around without sticking to other structures. These cells rely on van der Waals forces, which are considerably weaker than the forces measured by atomic force microscopy (AFM) and other typical methods of measuring adhesion. In fact, these adhesive forces are even weaker, than chemical bonds [82].

**Fig. 6:**
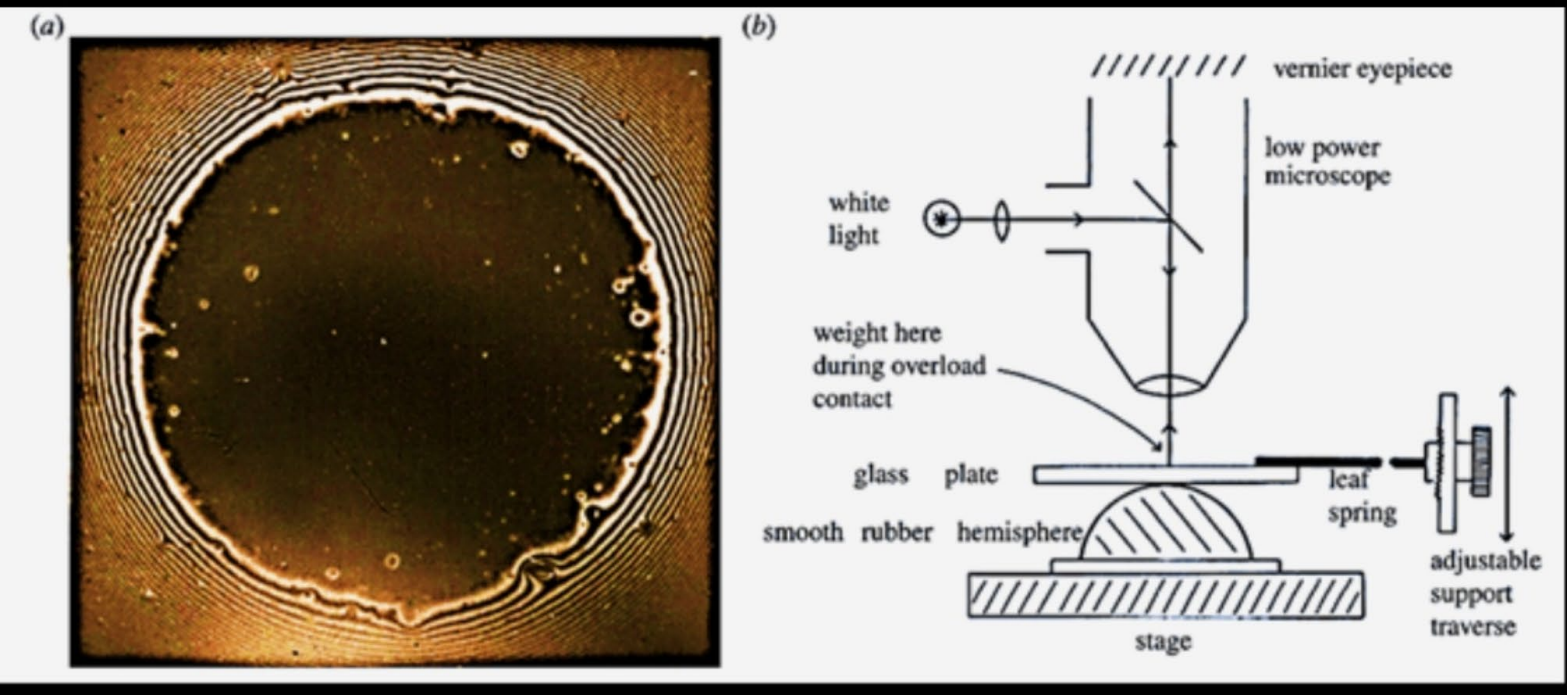
(**a**) Black contact spot of smooth rubber sphere viewed in microscope as shown in (**b**) [98]

In the late 1960s, Roberts devised a method for shaping a rubber sphere with an exceptionally sleek surface. This was achieved by applying pressure and cross-linking hot rubber material within a concave glass lens. When this impeccably smooth spherical rubber molding came into contact with another equally smooth sphere or a flat surface, it resulted in the formation of a black contact spot, as demonstrated in (Fig. 6) when observed under reflected light through a microscope. Typically, at zero load, a 1 mm radius black spot was observed, which could be enlarged by applying a normal force to further compress the surfaces. There were a few minor imperfections in the black spot attributed to dust particles, but the pliable nature of the rubber allowed it to conform around these flaws, rendering their impact negligible [83].

**Fig. 7:**
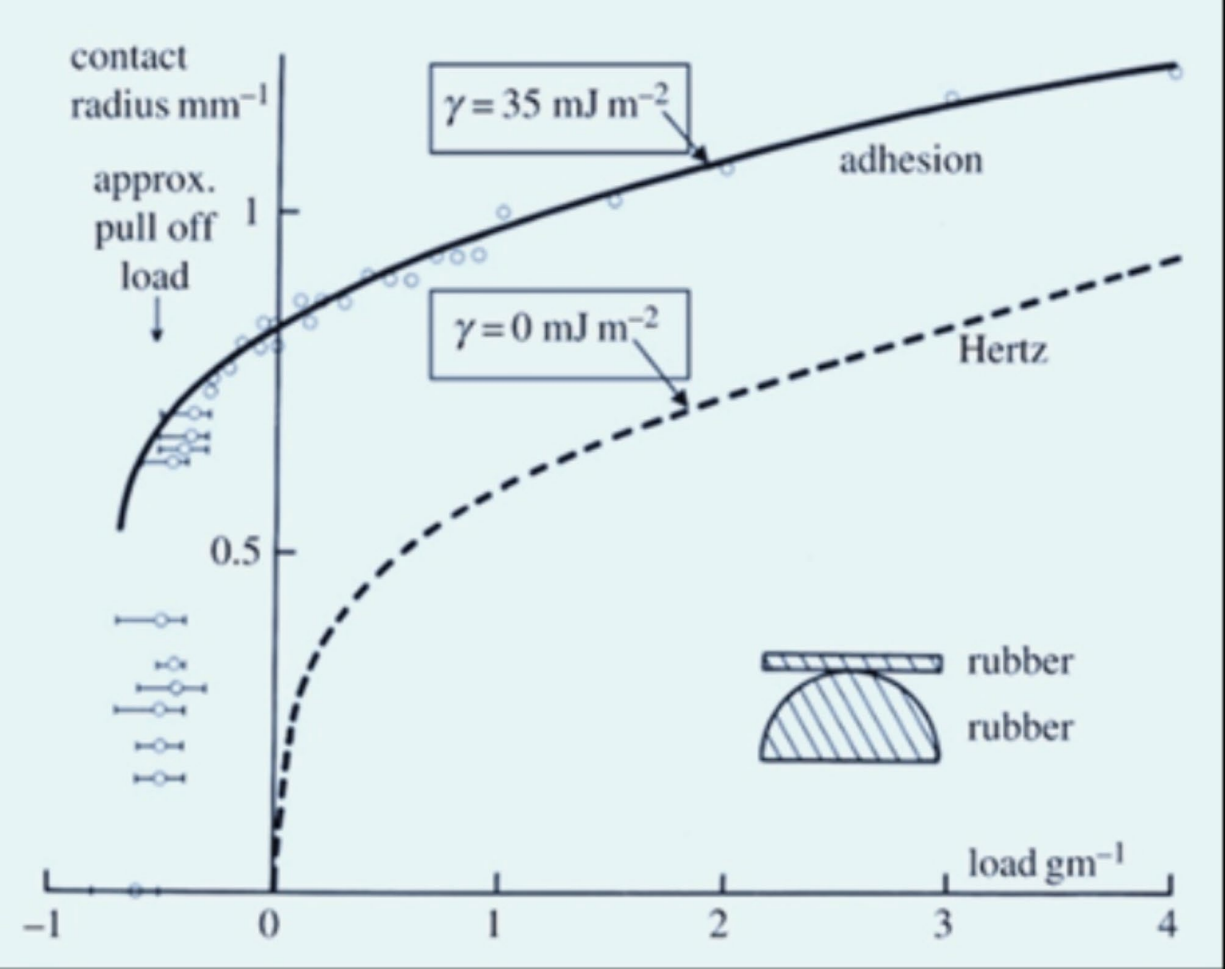
Smooth rubber adhesion results showing the contact circle radius as a function of normal applied force fitting JKR adhesion theory to compare with Hertz pure elastic contact. The adhesion pull-off force of 20.6 g was evident [98]

It proved intriguing that, in a clean and dry environment, the black spot’s size exceeded the predictions of the well-established Hertz theory of purely elastic contact, implying the presence of an adhesive force that attracted the surfaces toward each other. Furthermore, it became evident that a tensile force was required to separate the rubber surfaces and counteract this adhesive effect, as illustrated in ( Fig. 7) Despite taking meticulous precautions to eliminate any potential electrostatic or dipole forces, the adhesion phenomenon persisted [83].

The findings indicate that cell adhesion can be likened to the adhesion observed on smooth elastomer or gelatin spheres. Both theory and experimentation indicated that van der Waals forces were responsible for this adhesion on these materials. The factors influencing adhesion, such as contact diameter, elasticity, and sphere radius, were elucidated. Furthermore, the presence of water, led to a reduction in adhesion, with the latter contributing to a further decrease in adhesion strength that This prompts an inquiry into whether there is evidence supporting the existence of such forces between the retinal pigment epithelium and photoreceptors. The retinal pigment epithelium (RPE) consists of a single layer of cuboidal cells positioned between the photoreceptors and choriocapillaris. Its unique function is to engulf and repurpose the outer segment of photoreceptors, along with retinaldehyde, which serves as the chromophore for rhodopsin [84, 85]. RPE cells are unable to regenerate, and they must endure throughout one’s lifetime in order to preserve vision [86, 87]. The buildup of harm caused by exposure to light-induced damage in RPE cells is thought to contribute to the decline of RPE cells in the retina. Blue light, in particular, can be quite detrimental [81], and triggers the programmed cell death of rat RPE cells through a mechanism involving free radicals [88, 89]. While lipofuscin’s role in RPE cell loss due to exposure to blue light may be significant, it could particularly hold importance in aging RPE [90].Fetal RPE cells are also vulnerable to blue light-induced apoptosis (BLIA) [91]. From a metabolic perspective, RPE cells exhibit significant activity and possess a considerable amount of mitochondria. Exposure to blue light prompts the generation of reactive oxygen species (ROS) within the mitochondria of RPE cells and has been shown to lead to cell apoptosis, possibly triggered by ROS, which cause damage to mtDNA [92]. Furthermore, it has been proposed that the buildup of damage resulting from ROS over one’s lifetime does not only affect RPE cell loss but also plays a role in the aging process as a whole. Melanin is a diverse biological polymer found extensively in nature, and it is the sole known biopolymer that possesses inherent stable free radicals. Extra external free radicals can be reversibly generated in melanin when exposed to visible or UV radiation [93, 94].The building blocks of melanin are created through the oxidation of tyrosine, and various types of these building blocks can be combined to form natural melanin. However, melanin found in RPE is predominantly comprised of indole Quinone components. Melanin production in RPE takes place within melanosomes, resulting in the formation of melanin oligomers consisting of multiple monomers. At least four of these generate unique free radicals within melanin [95]. Melanin building blocks are linked together to form flat structures consisting of 4 to 6 monomers each. These flat structures then combine into larger aggregates through stacking and van der Waals interactions [96, 97]. Here we can see that van der walls interactions play a role on protection from BLIA so without these interaction this will lead to distraction of RPE induced by free radicals which caused by blue light.

**Fig. 8:**
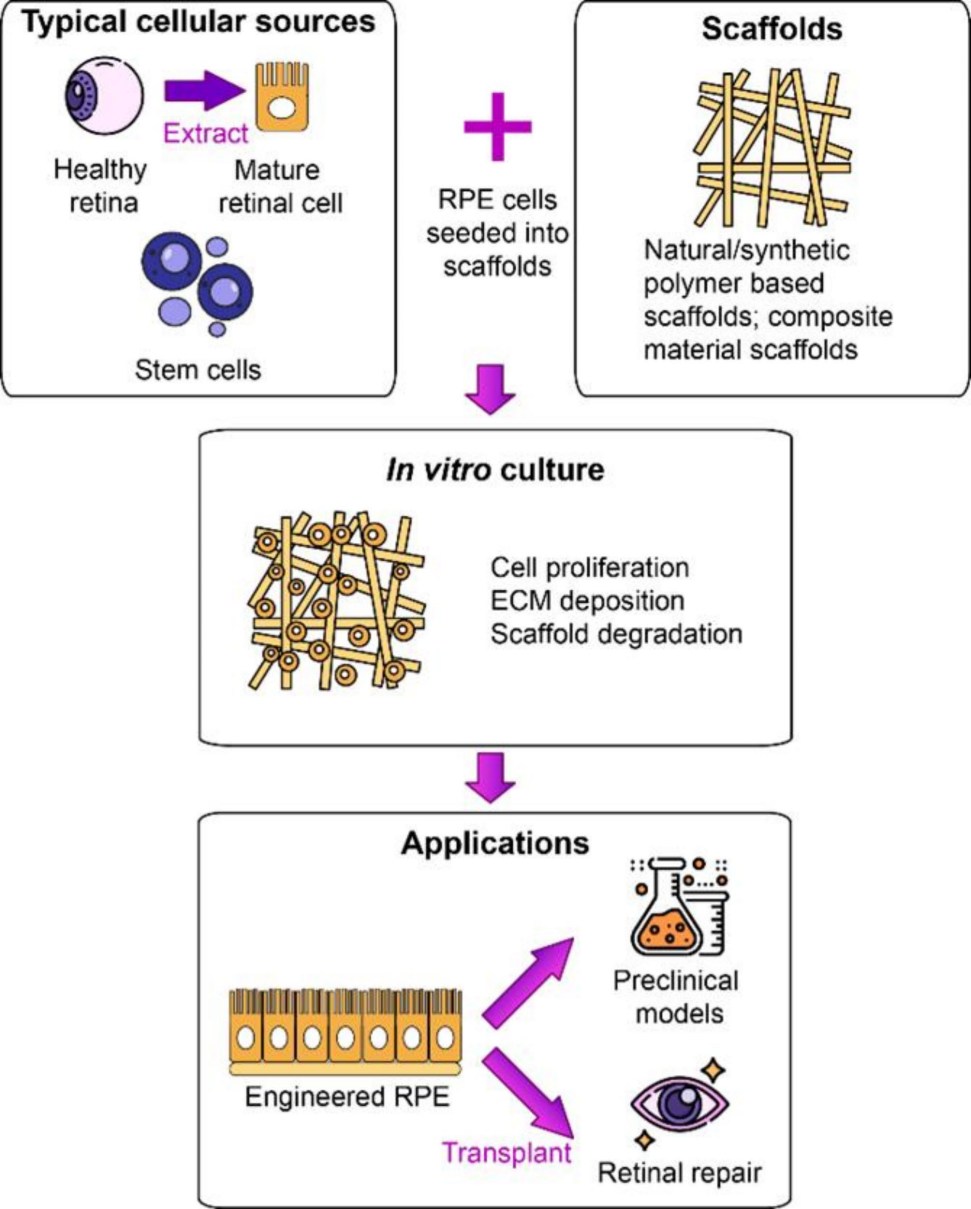
Scaffold-based tissue engineering

However, in the human retina, RPE cells have a hexagonal shape with an average height of approximately 10 μm [98]. Despite successful in vitro cultivation for over a century, RPE-related diseases remain poorly understood due to the complexity of these cells and the limitations of human models [99]. To advance new treatment approaches for RPE-related conditions, tissue engineering (TE) has been employed to construct preclinical models for drug testing and to investigate RPE mechanisms. TE is also used to generate RPE implants for retinal repair. Two methods, scaffold-free and scaffold-based, have been developed. Scaffold-free methods involve the regeneration of tissues through the creation of cell aggregates and sheets. For instance, cell sheet engineering utilizes cell growth and the secretion of its own extracellular matrix (ECM) to achieve the desired mechanical strength [100]. Scaffold-free methods often face challenges related to their susceptibility and vulnerability during surgical handling. Over the last decade, scaffolds have emerged as a promising approach to enhance cell-based RPE therapies. In this method, RPE cells are cultured on scaffolds and then transplanted beneath the retina, where they grow and mature into a functional monolayer. The core idea behind scaffold-based approaches is to create a three-dimensional (3D) environment for cell development by designing and producing scaffolds, both in laboratory settings and within living organisms [101].Typically, stem cells or cells obtained from healthy retinas are planted onto these scaffolds, with the anticipation that they will multiply and rejuvenate RPE tissue (Fig. 8) [102].

Designing scaffold structures to resemble the basement membrane (BM) has been demonstrated to enhance cell adhesion and facilitate cell growth. In contrast to chemical techniques, physical surface treatments like adsorption and coating are often more biocompatible, secure, and economical. Typically, scaffolds are immersed in solutions containing adhesive molecules, and the bonding between surface groups and scaffolds occurs through electrostatic, hydrophobic, van der Waals, and hydrogen bonding forces. Another prevalent approach to physical surface treatment involves the application of coatings onto organic materials [103]. Laminin, an extracellular matrix (ECM) protein, is frequently applied to coat scaffold surfaces. This enhances cell adhesion, promotes cell growth, encourages differentiation into mature retinal neurons, and boosts the expression of mature retinal proteins [104]. From this, we can see that these forces have indirectly contributed to the preservation of engineered RPE.

Exudative Retinal Detachment (ERD) occur when there is accumulation of fluids in the subretinal space between the retinal pigment epithelium and photoreceptors. however this space is narrow but under pathological conditions it can open because they disrupt BRB. occurrence of ERD and Breakdown of BRB is associated with conditions like inflammation, infection, neoplasm and degeneration.

### Etiology of exudative retinal detachment in the uveitis clinics 

Occurrence of ERD can result from inflammatory and infectious conditions through disrupting iBRB or oBRB or maybe both. infections can result from bacterial agents like tuberculosis and syphilis, viral infections like cytomegalovirus, fungal agents like Candida and Aspergillus, and nematodes like Toxocara and Cysticerca.

Non-infectious conditions are Vogt-Koyanagi-Harada syndrome, intermediate uveitis, sympathetic ophthalmia, posterior scleritis, and the white-dot syndromes [105]:

- **Vogt-Koyanagi-Harada (VKH) syndrome** is a multisystem disease that affects melanin containing tissues, such as choroid, skin,, ear and meninges [106].
- **Sympathetic ophthalmia (SO)** is characterized by bilateral granulomatous panuveitis that typically arises following ocular surgery or penetrating injuries. It typically manifests as panuveitis, showcasing granulomatous mutton-fat keratic precipitates, along with the presence of anterior chamber cells and flare, vitreous cells, and yellow-white lesions located in the periphery of the fundus [107].
- **Idiopathic orbital inflammatory syndrome (IOIS)**, also known as orbital pseudotumor, ranks as the third most prevalent orbital pathology, affecting approximately 5–10% of cases. It is typically diagnosed through exclusion and is believed to have an autoimmune etiology [108]. IOIS can affect many parts of the orbit, including the muscles, glands, fat, and optic nerve [109].
- **Serpiginous choroiditis** is a condition that result in bilateral and progressive inflammation in the choroid, with its cause remaining unknown, This inflammation leads to the loss of choriocapillaris and the atrophy of the retinal pigment epithelium (RPE), resulting in scarring [110, 111].
- **Acute posterior multifocal placoid pigment epitheliopathy (APMPPE)** is an inflammatory condition affecting the retina and choroid. It presents with sudden vision loss and the emergence of multiple flat, yellow-white lesions in the retinal pigment epithelium (RPE) and choriocapillaris. Depending on where these lesions occur, patients may experience central or side vision loss. Typically, the retina above these lesions looks normal [112].

**The Impact of Electrostatic Forces**,** Van Der Waals Forces**,** and Osmotic Pressure on Retinal Health: Pathophysiology and Clinical Consequences**.

A range of retinal diseases occurs because the barriers lose their functionality due to disrupted molecular interface forces such as electrostatic forces and van der Waals forces along with osmotic pressure. The retinal homeostasis needs these forces for maintaining stability while they work to preserve both structure and visual transmission. Retinopathological events form a sequence after fundamental force dysregulation, which results in retinal tissue death, vascular fluid loss, and vision impairment. These molecular forces maintain fundamental roles in the pathophysiology of the diseases described below and their effects on retinal function.

1. Diabetic Retinopathy (DR): Diabetic retinopathy, which causes blindness among working-age adults, features BRB breakdown caused by electrostatic interactions and van der Waals forces, which become dysfunctional in this condition. Continued high blood sugar levels of diabetes create advanced glycation end products (AGEs) that impair endothelial cell functions and weaken van der Waals forces between endothelial cells, thus damaging the BRB. The altered blood vessel permeability enables fluid lipids and proteins to leak into the retina and create retinal edema at the macula site termed as diabetic macular edema (DME). The retina develops ischemic areas as a result of which neovascularization occurs through new fragile blood vessel growth because of oxygenation problems. New vessel growth in the retina has a high tendency to break open, resulting in bleeding that damages the retina even more. The retina experiences the development of edema because of osmotic pressure changes that combine with impaired fluid regulation mechanisms [113].
2. Age-Related Macular Degeneration (AMD): In AMD, both osmotic pressure imbalances and van der Waals force dysfunction play significant roles in disease progression. The dry form of AMD results in drusen accumulation (yellow deposits) between retinal pigment epithelium (RPE) cells and the choroid tissue. The osmotic equilibrium disruption from accumulated deposits prevents the RPE from properly managing fluid balance and waste material elimination. The inflammation together with oxidative stress affects RPE functionality until the RPE becomes dysfunctional, thus speeding up retinal degeneration. Choroidal neovascularization occurs as a result of impaired van der Waals forces, which leads to the formation of abnormal blood vessels beneath the retina in the wet form of AMD. Leaky new vessels in this condition produce subretinal fluid collections that result in macular edema, retinal detachment, and continuous worsening of central vision. The retina’s osmotic changes make fragile vessels release more fluids, leading to damage of the retinal structure, which worsens vision loss [114].
3. Retinitis Pigmentosa (RP): Patients with retinitis pigmentosa, which belongs to the inherited retinal dystrophies, experience electrostatic force disruption between photoreceptors and RPE cell functions in the retina. The photoreceptors slowly degenerate because they fail to maintain electrostatic forces that control ion exchange throughout photoreceptor cells, resulting in faulty phototransduction. The damaged photoreceptor outer segments fail to undergo efficient phagocytosis by the RPE because of force dysfunction, which leads to toxic waste buildup in the retina, thus promoting photoreceptor degeneration. The condition causes vision deterioration, which begins with the loss of night vision and peripheral vision before ultimately causing deterioration of central vision. Disease advancement occurs rapidly because electrostatic bond breakdown combined with damaged retinal structure hinders self-repair mechanisms in the retina [115].
4. Retinal Vein Occlusion (RVO): A blockage of any retinal vein causes increased venous pressure and breakdown in osmotic pressure regulation throughout the retinal vessels. Enhanced blood vessel pressure makes retinal blood vessels leak fluid, which causes macular retinal edema that creates blurred and distorted vision. The pathological process in RVO becomes worse due to osmotic force abnormalities because disrupted venous blood flow causes retinal tissue fluid accumulation, leading to worsened vision. New fragile blood vessels that form because of venous occlusion-induced ischemia raise the chance of bleeding problems and create additional macular edema.
5. Central Serous Chorioretinopathy (CSCR): Osmotic pressure disturbances in CSCR lead to the formation of subretinal fluid that causes its development. The leakage of fluid between choroidal circulation and subretinal space results in serous retinal detachment. The osmotic pressure irregularity generates fluid accumulation that develops between the RPE and retina and produces vision distortion in the central field. The choroidal circulation malfunction seems to increase osmotic gradients, which produce fluid leakage in CSCR. Regular resolution of this condition happens independently, yet persistent cases might result in irreparable retinal damage together with visual impairment. Belief exists that osmotic pressure generates fluid accumulation, which elsewhere triggers retinal detachment in people suffering from CSCR, thus demonstrating why osmotic equilibrium maintenance becomes essential for retinal health.
6. Uveitis: Uveitis leads to uveal tract inflammation because it triggers complex immune system reactions with retinal vasculature components. The pathophysiological mechanism of uveitis depends on endothelial cell difficulties in electrostatic and van der Waals bond maintenance, which leads to blood-retinal barrier breakdown. The vasculature permeability rate increases in the retinal tissue because of these disruptions, causing inflammatory cells along with proteins to penetrate the tissue. Such occurrences lead to the development of retinal edema, vascular leakage, and macular edema that create vision impairment. The osmotic stress from this fluid leakage causes the retina to swell additionally, which subsequently leads to both retinal scarring and neovascularization as complications. Uveitis may progress in its most serious stage to create retinal ischemia, which causes permanent blindness.
7. Macular Telangiectasia (MacTel): Various medical professionals identify Macular telangiectasia as a disease which creates telangiectatic vessels that leak fluids into the retina. The blood vessel structural failure caused by weakened van der Waals forces leads to formation of these leak-prone fragile abnormal vessels. The fluid gathers inside the macula because of this process thus forming macular edema which ends in central vision loss. Visual function deteriorates from the osmotic imbalance created by fluid leakage through these fragile abnormal vessels. The progressive nature of MacTel tends to cause vision deterioration but permanent central vision loss results from inadequate management of this condition [113].
8. Stargardt Disease: The progressive degeneration of the macula, which causes lipofuscin buildup in retinal pigment epithelium, occurs as a result of Stargardt disease. The breakdown of RPE-photoreceptor electrostatic forces and van der Waals interactions creates an incapable condition for normal cell debris removal. Critical photoreceptor loss in the macular area results from oxidative stress along with retinal inflammation caused by this degenerative process. The deposition of lipofuscin creates problems for RPE cell activities that eventually result in macular atrophy and central vision loss. This genetic disorder starts appearing in early childhood or adolescence and mainly damages the macula yet progresses until all central vision fades away.
9. Cone-Rod Dystrophy: Cone-rod dystrophy is a group of retinal disorders because it causes progressive vision loss through photoreceptor cone and rod degeneration. Pathogenesis of the disease advances because retinal cell signaling and photoreceptor maintenance become disrupted by van der Waals and electrostatic forces. When forces between phototransduction components are interrupted, the photoreceptor cells become damaged through oxidation until they degenerate. Advanced stages of this condition result in the loss of photoreceptor cells, which causes vision loss in central vision as well as peripheral vision loss. Most patients become affected by this inherited genetic disorder at any stage of life based on their individual mutations [115, 116].

The successful operation of the retina depends entirely on the precise biosystem consisting of electrostatic forces, van der Waals forces, and osmotic pressure. Many retinal diseases emerge when these forces become disrupted, so the disease list includes diabetic retinopathy and age-related macular degeneration alongside retinitis pigmentosa, retinal vein occlusion, central serous chorioretinopathy, uveitis, macular telangiectasia, Stargardt disease, and cone-rod dystrophy. Understanding the molecular mechanisms underlying these diseases can provide valuable insights into potential therapeutic targets and help in developing strategies to preserve retinal health and prevent vision loss.

## Conclusion

The intricate interplay of electrostatic forces, van der Waals forces, and oncotic pressure within the retina’s microenvironment, particularly in the context of the retinal pigment epithelium (RPE) and photoreceptor interactions. The complexity of ocular biology presents exciting opportunities for research and therapeutic interventions in ophthalmology.Electrostatic forces, manifested through the apical microvilli of the RPE, serve as critical anchors and protectors, preventing the detachment of the photoreceptor layer and facilitating various vital processes within the subretinal space. The composition of the interphotoreceptor matrix (IPM) and the presence of molecules like Retbindin further modulate these forces, impacting processes such as visual pigment regeneration and metabolite exchange.In the realm of retinal tissue engineering, electrostatic forces are harnessed to optimize nutrient supply to transplanted RPE cells. Techniques involving frame-supported ultrathin electrospun polymer membranes show promise in enhancing cell retention and promoting multicellular aggregate formation within the subretinal space. Scaffold-based strategies, leveraging physical surface treatments and interactions driven by electrostatic, hydrophobic, van der Waals, and hydrogen bonding forces, offer innovative approaches to RPE tissue regeneration.Oncotic pressure, primarily driven by proteins like albumin, plays a crucial role in maintaining the blood-retina barrier (BRB), safeguarding the retinal microenvironment from harmful substances while ensuring proper nutrient exchange. The delicate balance between hydrostatic and oncotic pressures within the eye’s vasculature is essential for preserving retinal health.Van der Waals forces, though weaker than covalent bonds, contribute to cell adhesion, and their presence has been observed in the interaction between smooth elastomer surfaces. While their specific role in retinal interactions warrants further investigation, it’s evident that these forces play a role in cell adhesion and should be considered in the context of retinal health.Overall, the multifaceted interplay of these forces reveals the remarkable intricacies of retinal biology and presents avenues for innovative therapeutic interventions. Understanding and harnessing these forces may hold the key to advancing treatments for retinal disorders and preserving vision. As we delve deeper into this fascinating realm of ocular biology, we can look forward to breakthroughs that benefit patients and researchers alike.

## Data Availability

No datasets were generated or analysed during the current study.

## Declarations

### Method of literature

Explore scientific articles and research papers on Minimally Invasive Glaucoma Surgery (MIGS) in Google Scholar and pubmed as a transformative approach to glaucoma management. Investigate MIGS techniques, classifications, indications, contraindications, devices, and their impact on patient outcomes, with a focus on precision, safety, and minimal invasiveness. Assess the comparative advantages of MIGS over traditional glaucoma treatments, including reduced complications and improved patient comfort. Examine clinical cases and studies showcasing the efficacy of MIGS in various types of glaucoma. Delve into the potential of MIGS to change the landscape of ophthalmology and enhance the quality of life for glaucoma patients.
